# Linear Discriminant Analysis for Investigating Differences in Upper Body Movement Symmetry in Horses before/after Diagnostic Analgesia in Relation to Expert Judgement

**DOI:** 10.3390/ani12060762

**Published:** 2022-03-17

**Authors:** Thilo Pfau, David M. Bolt, Andrew Fiske-Jackson, Carolin Gerdes, Karl Hoenecke, Lucy Lynch, Melanie Perrier, Roger K. W. Smith

**Affiliations:** 1 Department of Clinical Science and Services, The Royal Veterinary College, Hawkshead Lane, North Mymms, Hatfield AL9 7TA, UK; dbolt@rvc.ac.uk (D.M.B.); afiskejackson@rvc.ac.uk (A.F.-J.); khoenecke7@rvc.ac.uk (K.H.); llynch6@rvc.ac.uk (L.L.); mperrier@rvc.ac.uk (M.P.); rksmith@rvc.ac.uk (R.K.W.S.); 2 Faculty of Veterinary Medicine, University of Calgary, 2500 University Dr NW, Calgary, AB T2N 1N4, Canada; 3 Faculty of Kinesiology, University of Calgary, 2500 University Dr NW, Calgary, AB T2N 1N4, Canada; 4 Pferdeklinik Hochmoor GmbH, D-48712 Gescher, Germany; c.gerdes@pferdeklinik-hochmoor.de

**Keywords:** horse, lameness, diagnostic analgesia, straight-line, circular movement, linear discriminant analysis

## Abstract

**Simple Summary:**

Identifying the anatomical structures involved in causing pain and an associated lameness in a horse typically requires assessment in straight lines and circles and using regional administration of local anesthetic drugs (diagnostic analgesia). Visual assessment of changes in movement are affected by bias, i.e., expected changes influence decisions. Quantitative measurements with inertial sensors aim at removing this bias. The current study is aimed at investigating how a specific data-driven method, linear discriminant analysis (LDA), may be useful for aiding veterinary decision making about perceived changes in lameness. Changes in movement data after diagnostic analgesia and expert judgements from 53 lame horses were used to study (a) the accuracy of LDA-based decision making, (b) differences between straight-line and circular movement and (c) which commonly used movement features are most useful in this context. Accuracy was comparatively low and varied considerably between 36% and 57%, indicating considerable overlap between movement symmetry data of the diagnostic analgesia categories. The best data-driven separation between categories was observed when the limb in which perineural anaesthesia had been performed was on the inside of the circle (on hard ground for forelimb and on soft ground for hindlimb diagnostic analgesia). Movement features of all three landmarks (head, withers, pelvis) were important for data-driven classification, emphasizing the complexity of the movement pattern changes after diagnostic analgesia observed in lame horses.

**Abstract:**

Diagnostic analgesia and lunging are parts of the equine lameness examination, aiding veterinarians in localizing the anatomical region(s) causing pain-related movement deficits. Expectation bias of visual assessment and complex movement asymmetry changes in lame horses on the lunge highlight the need to investigate data-driven approaches for optimally integrating quantitative gait data into veterinary decision-making to remove bias. A retrospective analysis was conducted with inertial sensor movement symmetry data before/after diagnostic analgesia relative to subjective judgement of efficacy of diagnostic analgesia in 53 horses. Horses were trotted on the straight and on the lunge. Linear discriminant analysis (LDA) applied to ten movement asymmetry features quantified the accuracy of classifying negative, partial and complete responses to diagnostic analgesia and investigated the influence of movement direction and surface type on the quality of the data-driven separation between diagnostic analgesia categories. The contribution of movement asymmetry features to decision-making was also studied. Leave-one-out classification accuracy varied considerably (38.3–57.4% for forelimb and 36.1–56.1% for hindlimb diagnostic analgesia). The highest inter-category distances (best separation) were found with the blocked limb on the inside of the circle, on hard ground for forelimb diagnostic analgesia and on soft ground for hindlimb diagnostic analgesia. These exercises deserve special attention when consulting quantitative gait data in lame horses. Head and pelvic upward movement and withers minimum differences were the features with the highest weighting within the first canonical LDA function across exercises and forelimb and hindlimb diagnostic analgesia. This highlights that movement changes after diagnostic analgesia affect the whole upper body. Classification accuracies based on quantitative movement asymmetry changes indicate considerable overlap between subjective diagnostic analgesia categories.

## 1. Introduction

A major component of the equine veterinary lameness examination is the use of diagnostic analgesia, i.e., perineural (“nerve blocking”) and/or intra-synovial (“joint blocking”) anesthesia, with the aim of providing information that will aid the decision making about which part(s) of a limb is (are) experiencing discomfort during locomotion [1]. In non-verbal patients (animals) this is particularly important for guiding diagnostic imaging in terms of which anatomical region to focus on and which technology to choose (e.g., focusing on soft tissue versus bony structures) and thus influencing treatment options. Relating the visual appearance of a lameness to findings from palpation or diagnostic imaging is crucial in order to be able to judge the ‘functional’ relevance of any identified abnormalities [2].

In addition to diagnostic analgesia, diagnosis of the source of pain causing a lameness is assisted by exercising the horse under different conditions, e.g., in circles on hard and soft ground [3,4]. Clinical experience indicates that the origin of a lameness may result in more or less severe changes in relation to the affected limb positioned on the inside or outside of the circle and/or to the surface characteristics [3]. The first may be related to the difference in extra-sagittal angulation and/or force between the inside and the outside limb [5,6] and/or the amount of body lean [7]. The second may be related to differences in impact accelerations/vibrations [8,9,10], in stance time, stride time or duty factor [10] and consequently peak force [11,12] and/or changes in breakover [13].

In the lameness examination, both techniques—diagnostic analgesia and circular exercise on the lunge—are often combined. Studies with quantitative gait analysis have established guideline values for the amount of head (6 mm) and pelvic (3 mm) movement asymmetry [14], which, when exceeded in a horse presented to a veterinarian, can be used to identify the most likely limb causing the lameness. Importantly, these movement asymmetries have also been associated with force imbalances [15,16]. Adding quantitative assessments relating to the effects of diagnostic analgesia may help with reducing the amount of expectation bias that has been documented for visual decision making [17] and assist in determining partial improvements confidently, overcoming the limited sensitivity of the human visual system when assessing movement symmetry [18]. Previous quantitative data from lunge exercise have provided information about variations between repeat measurements [19]; the expected changes as a function of speed, surface and circle radius [20,21]; changes after induction of lameness [22] and associations between asymmetries on the lunge with pre-existing movement asymmetries measured during straight line locomotion [23,24]. However, relevant studies with quantitative gait data combining diagnostic analgesia with lunge exercise are lacking.

The aim of this study was to investigate the discriminative power of upper body movement symmetry differences calculated before and after administration of diagnostic analgesia for differentiating between blocks judged subjectively according to their efficacy by experienced veterinarians during a lameness exam. The study objectives were to quantify differences in upper body movement symmetry before and after diagnostic analgesia for different exercise conditions typically encountered during the lameness examination, such as straight-line trot and trot in circles on soft and hard ground and to categorize block efficacy as ‘negative’ (subjectively no improvement: 0–30% [25]), ‘partially positive’ (subjectively some improvement but with residual lameness: >30–70% [25]) and ‘positive’ (subjectively lameness resolved or switched to different limb: >70–100% [25]). This was achieved by consulting the case records and applying a data-driven feature transformation and classification method—linear discriminant analysis, which has been successfully applied to quantitative data collected in horses with hindlimb lameness [26]—to provide evidence relevant for investigating three hypotheses:

1. The accuracy of a data-driven categorization of the efficacy of diagnostic analgesia from quantitative gait changes would reflect that increased uncertainties exist when scoring hindlimb lameness (increased intra- and inter-observer variability for scoring hindlimb than for scoring forelimb lameness [27,28]) and would hence provide higher accuracy for categorizing forelimb and lower accuracy for hindlimb diagnostic analgesia.
2. Quantitative distances between data clusters, as a measure of the data-driven ability to differentiate between categories of block efficacy, would vary across exercise conditions. For example, we expect lunge exercises with the blocked limb on the inside of the circle to provide higher distances between diagnostic analgesia categories, i.e., a better classification, due to the documented increase in movement asymmetry for that exercise condition in horses with pre-existing straight-line movement asymmetries [21,24].
3. In analogy to visual observation, quantitative features derived from head movement would be most useful for differentiating between forelimb diagnostic analgesia efficacy categories, and pelvic movement asymmetry features would be most useful for differentiating between hindlimb categories.

## 2. Materials and Methods

This retrospective study was approved by the Royal Veterinary College Social Sciences Research Ethics Board with the unique reference number (SR2020-0250).

### 2.1. Horses and Facilities

Inertial measurement unit (IMU) gait analysis data of 53 horses were evaluated retrospectively from hospital records of clinical gait analysis examinations at two veterinary referral hospitals (Royal Veterinary College (RVC) Equine Referral Hospital, United Kingdom, N = 39; Pferdeklinik Hochmoor GmbH, Germany, N = 14). Measurements had been obtained for each horse in trot, on a straight line on a hard surface and during circular exercise to the left and right on a soft surface, a hard surface, or both. The hard surface at one location was a non-slip coated tarmac and at the other a paving stone surface. The soft surface consisted of a sand/fiber mixture at one location and of a sand-based arena surface at the other. Circular exercise was performed with radii during lunging (or circular movement in-hand where deemed appropriate) ranging between 5 m and 10 m.

All horses had been referred for clinical investigation of lameness and/or poor performance by their primary care veterinarian. There were 26 Warmblood or Warmblood X, seven Thoroughbred or Thoroughbred X, five Irish Sport horses, four riding ponies, three cobs, two Quarter horses, and one each of British Sport horse, Shire X, Irish Draft X and Spanish, as well as two horses of unknown breed. Average age of the horses was 10.4 years (median 11, range 3–20). Thirty-one of the horses were geldings, 21 were mares and there was one stallion.

### 2.2. Instrumentation

Vertical displacement data were collected by attaching five to eight MTw (Xsens, Enschede, The Netherlands) IMUs (dimensions 47 × 30 × 13 mm, mass 16 g) placed at the: poll, withers, sacrum, left and right tuber coxae (5-sensors) and additionally at the thirteenth thoracic vertebra, first lumbar vertebra and above the tail root (8-sensors). In this study, only data from the 5-sensor setup were evaluated. Raw sensor data (tri-axial acceleration, tri-axial rate of turn and tri-axial magnetic field strength) were transmitted wirelessly at update rates of 60 to 100 Hz (see [29] for influence of sample rate) per individual data channel via a proprietary protocol (Xsens) to a receiver station (Awinda, Xsens) connected to a laptop computer running operating system Windows 10 (Pro, 64 bit, Microsoft, Redmond, WA, USA) communicating with MTManager (Xsens) software providing calibrated sensor data and orientation data.

### 2.3. Movement Symmetry Parameters

Using MATLAB user interface scripts (R2015b, The Mathworks Inc., Natick, MA, USA), data were further processed by calculating movement asymmetry parameters for each trot stride based on vertical displacement calculated based on rotating calibrated tri-axial acceleration data into a horse- and gravity-based reference frame via sensor orientation and knowledge of alignment of the sensor x-axis with the horse’s sagittal axis following published protocols [30,31]. Stride segmentation was defined by the sacrum sensor using published methods [32], and median values were calculated for previously described movement asymmetry parameters quantifying differences in head displacement (HD), withers displacement (WD) and pelvic displacement (PD) minima (min), maxima (max) and upward movement amplitudes (up) between stride halves: HDmin, HDmax, HDup, WDmin, WDmax, WDup, PDmin, PDmax, PDup. Additionally, the hip hike difference was calculated comparing left and right tuber coxae movement amplitudes during contralateral stance [33].

#### 2.3.1. Sign Convention

For all movement parameters, asymmetries typically encountered in left forelimb (head, withers) or left hindlimb (pelvis) lame horses were assigned negative values and asymmetries typically encountered in right forelimb (head, withers) or right hindlimb (pelvis) lame horses were assigned positive values.

#### 2.3.2. Normalization

Differences were then calculated between the condition before and after diagnostic analgesia. In order to be able to combine data from diagnostic analgesia administered to left limbs with data from diagnostic analgesia administered to right limbs without the opposite signs masking potential effects, data were normalized to reflect administering diagnostic analgesia to right limbs, by multiplying by −1 (inverting) movement asymmetry differences calculated from left limb diagnostic analgesia.

### 2.4. Exercise Conditions

In addition to the type of surface (soft or hard), normalized movement asymmetry differences before/after diagnostic analgesia were labelled according to the exercise from which they had been calculated as follows: straight line, inside rein, outside rein, inside/outside average rein. Lunge direction was expressed in relation to the positioning of the limb to which the diagnostic analgesia had been applied as “inside” for horses on the left rein with left limb diagnostic analgesia (or on the right rein with right limb diagnostic analgesia) and as “outside” for horses on the left rein after right limb diagnostic analgesia or on the right rein after left limb diagnostic analgesia.

### 2.5. Block Efficacy

Perceived outcome of diagnostic analgesia “efficacy” was subjectively determined by clinician observation during the lameness examination. Where lameness examinations had been conducted over multiple days, new “baseline” asymmetry values were collected at the beginning of each day to which differences were calculated for the first block on that day, and each subsequent block was assessed against its preceding condition, i.e., the first block compared to baseline gait data, the second block compared to the first block, and so on.

Block outcomes were categorized retrospectively based on consulting electronic case records. These included statements about the efficacy of the performed blocks in three subjective categories: “negative”, “partially positive” or “positive”, or statements about the subjective ‘percentage change’ after diagnostic analgesia which were in consultation with the clinicians mapped into the three categories as follows: “negative”, 0–30% [25]; “partially positive”, >30–70% [25]; “positive”, >70–100% [25]. The chosen retrospective procedure means that the subjective categorization does not specifically differentiate the efficacy of a block for each exercise (for each surface/direction combination) but is a generalized, subjective judgement about the efficacy across exercises. This general block efficacy category was assigned ‘as a global subjective judgement’ to all exercise conditions under which the horse had been observed for the specific diagnostic block and for which quantitative gait data was available.

**Figure 1:**
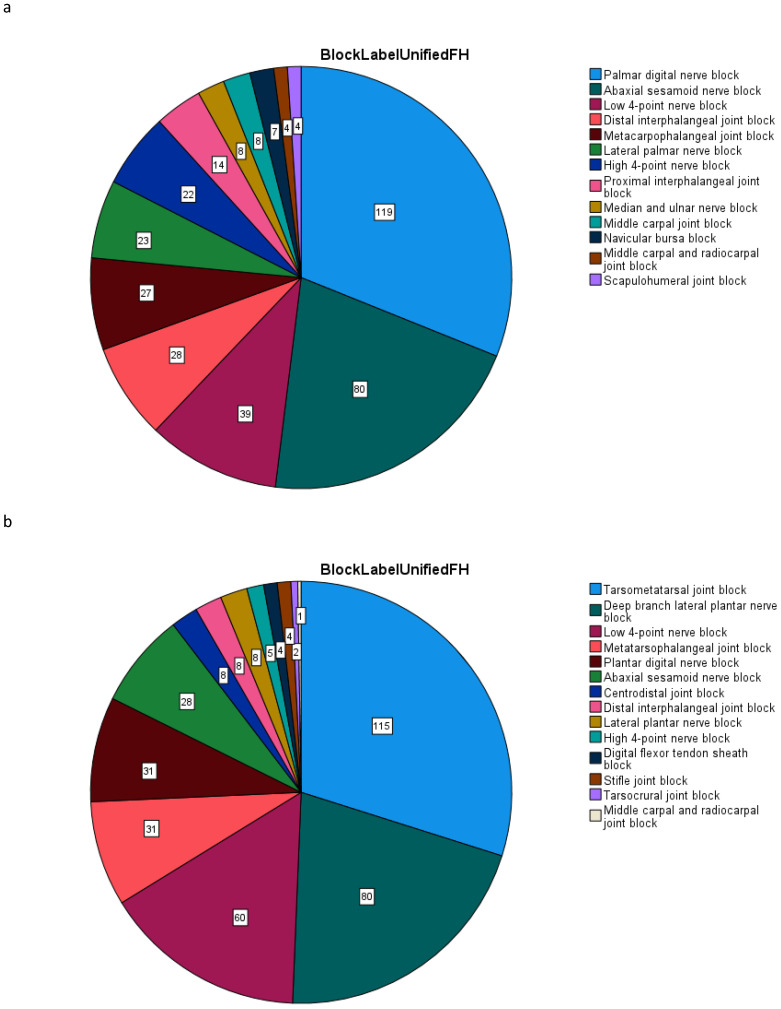
Frequency distribution of forelimb and hindlimb datasets of movement asymmetry differences calculated before/after diagnostic analgesia (DA), subdivided by the specific diagnostic blocks. (**a**) forelimb DA; (**b**) hindlimb DA. Further details about blocks: abaxial sesamoid nerve block: palmar/plantar nerves blocked at the level of the base of the proximal sesamoid bone; Low 4-point nerve block: palmar and palmar metacarpal nerves (forelimb), plantar and plantar metatarsal nerves (hindlimb) blocked just above the metacarpophalangeal joint (forelimb) or just above the metatarsophalangeal joint (hindlimb); High 4-point nerve block: palmar and palmar metacarpal nerves (forelimb), plantar and plantar metatarsal nerves (hindlimb) blocked just below the carpometacarpal joint (forelimb) or just below the tarsometatarsal joint (hindlimb); centrodistal joint block, also referred to as distal intertarsal joint block.

Straight line data of a total of 86 instances of forelimb and 88 instances of hindlimb diagnostic analgesia were included in the dataset. Blocks performed included perineural nerve blocks, as well as intra-articular and intrathecal intra-synovial techniques. A frequency distribution of the forelimb and hindlimb datasets with respect to the specific blocks administered can be found in Figure 1, with additional information about the corresponding exercise conditions in Table S1. In the context of the LDA method and the normalized features based on difference values calculated from conditions directly before and after diagnostic analgesia, one of the focal points of this first study is the identification of features that are useful across a number of potential sources of variation (clinicians, horses, source(s) of lameness etc.) rather than a detailed analysis of all potential sources of variation.

### 2.6. Linear Discriminant Analysis

Linear discriminant analysis (LDA), a method that has been successfully employed previously to categorize data from horses with hindlimb lameness [26], was employed in the present study to investigate how well the ten quantified movement asymmetry parameters were able to differentiate between the three subjective block efficacy categories.

LDA is a method that has been investigated and used in the context of multidimensional pattern recognition applications, such as speech recognition [34,35], and is an extension to principal component analysis with particular focus on differentiation between discrete categories in the face of sources of variation. In speech recognition, the categories are typically small units of speech (phonemes), and the sources of variation are, for example, different speakers or different accents or dialects. Here, the categories are the subjective judgements of the outcome of the block (block efficacy) and the sources of variation are the different clinicians, different horses with different cause(s) of lameness, different blocks or different exercise characteristics such as, for example, lunge radii.

In this study, the complete dataset was first subdivided into two subsets: data for forelimb and data for hindlimb diagnostic analgesia. Furthermore, separate LDA feature transformations were implemented for straight-line data, for inside rein, outside rein and average values calculated between the two reins [36]. A further subdivision was implemented by surface type (hard/soft). This resulted in twenty LDA transformations; ten for forelimb and ten for hindlimb diagnostic analgesia: straight line (hard surface), average rein (overall, soft, hard), inside rein (overall, soft, hard) and outside rein (overall, soft, hard). SPSS (version 28.0.0.0 (190)) was used for implementing all LDA transformations.

#### 2.6.1. Classification Accuracy

The performance of each LDA transformation was assessed by calculating reclassification accuracy and leave-one-out cross-validation accuracy (each case classified by the functions derived from all cases other than that case) from confusion matrices for the three block efficacy categories.

#### 2.6.2. Variation between Exercises

In order to evaluate how specific exercise conditions might influence LDA classification, centroid distances between the three block efficacy categories were calculated in the LDA-transformed feature space along the two canonical discriminant functions. The larger the differences between block efficacy category centroids, the better the data-driven separation. Individual distances between all three two-category pairings were calculated (negative versus partially positive, negative versus positive, partially positive versus positive) as well as the sum of the distances as an overall indicator of across-category separation. Scatter plots in the LDA-transformed feature space were created, illustrating the location of each individual diagnostic analgesia data point (i.e., of each investigated diagnostic block) as well as category centroids for the three efficacy categories in the transformed feature space.

#### 2.6.3. Movement Asymmetry Parameters

Standardized canonical discriminant function coefficients for the first and second function were tabulated according to the exercise condition (combination of movement direction and surface). The contribution of each LDA input feature, i.e., the weighting of each movement asymmetry parameter within each discriminant function, was quantified in terms of the absolute value. This contribution was illustrated via bar graphs (created in Microsoft Excel) for each discriminant function of each exercise condition. In order to allow reproducing the LDA transformation from the absolute values given in the results tables, originally positive values within the discriminant function are labelled “+ve”, and originally negative values are labelled “−ve”.

## 3. Results

In total, 768 feature sets containing all ten movement asymmetry parameter differences measured before and after diagnostic analgesia in 53 horses investigated for lameness/poor performance were analysed in this retrospective study. The case notes of five experienced veterinary specialists were accessed retrospectively and consulted for notes about the efficacy of individual diagnostic blocks categorized as ‘negative’, ‘partially positive’ or ‘positive’.

### 3.1. Classification Accuracy

**Figure 2:**
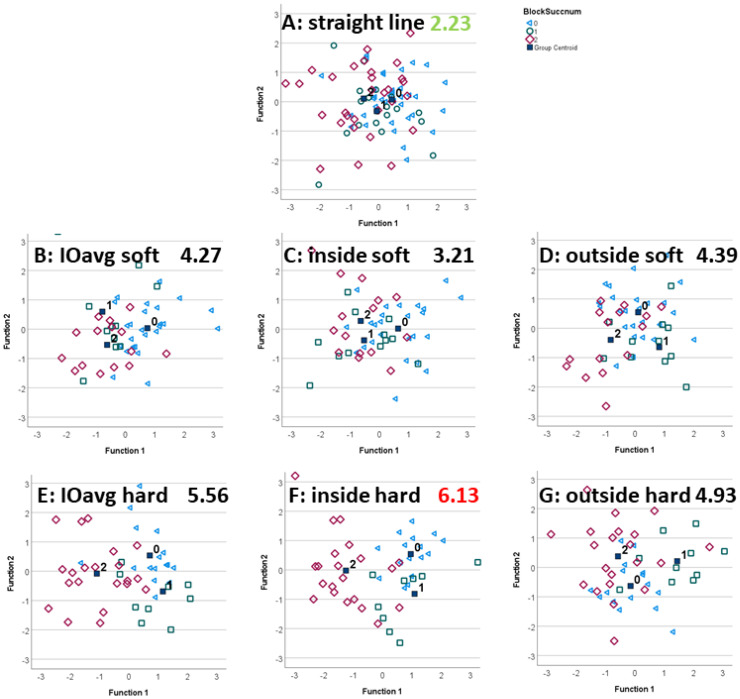
Scatter plots of the first (Function1, x-axis) and second (Function2, y-axis) standardized canonical discriminant function calculated from linear discriminant analysis (LDA) applied to movement symmetry differences before/after forelimb diagnostic analgesia (negative (0, blue triangle), partially positive (1, green square) and positive (2, red diamond)). Black filled squares indicate group centroids of the three category clusters. The highest value, the best separation between subjectively judged diagnostic analgesia efficacy categories is indicated in red, and the lowest values, worst separation, is indicated in green.

**Figure 3:**
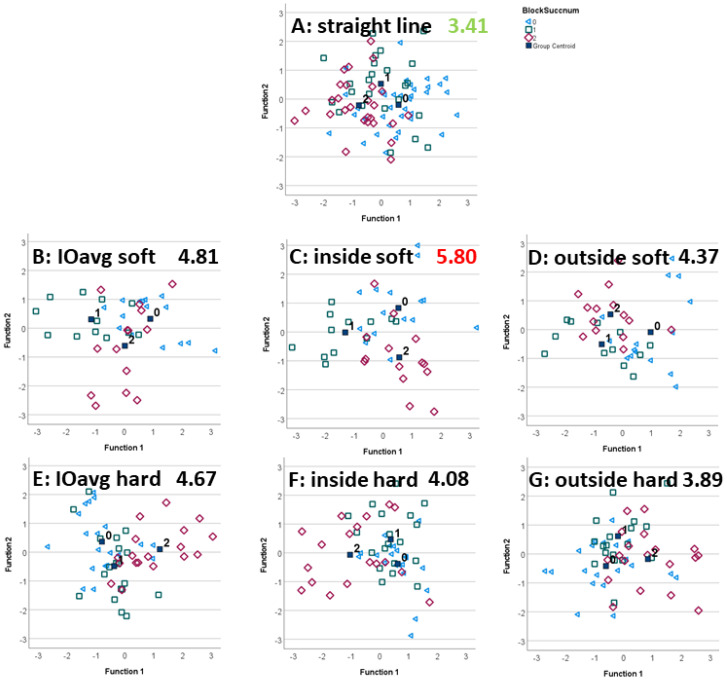
Scatter plots of the first and second standardized canonical discriminant function calculated from LDA applied to movement symmetry differences before/after hindlimb diagnostic analgesia (negative (0, blue triangles), partially positive (1, green square) and positive (2, red diamond)). Black filled squares indicate group centroids of the three category clusters. The highest value, the best separation between subjectively judged diagnostic analgesia efficacy categories is indicated in red, and the lowest values, worst separation, is indicated in green.

LDA reclassification and leave-one-out classification confusion matrices are reported in Table 1. Figure 2 (forelimb diagnostic analgesia) and Figure 3 (hindlimb diagnostic analgesia) present scatter plots of the two standardized canonical discriminant functions as the x- and y-axis with individual data points (individual blocks) and diagnostic analgesia efficacy category centroids.

Table 1 provides an overview of the reclassification and leave-one-out classification accuracies for LDA classification based on different subsets of the data subdivided by diagnostic analgesia location (front or hindlimb), exercise (straight-line, inside rein, outside rein, average) and surface (soft or hard).

For forelimb diagnostic analgesia, the highest leave-one-out LDA-classification accuracy (57.4%) was achieved with the data from the lunge and the horse trotting with the blocked limb on the inside of the circle on hard ground, followed by exercise with the blocked limb on the outside of the circle on soft ground (55.8%). The lowest leave-one-out classification with 38.3% was found for the outside rein data on the hard surface followed by the straight-line data with 39.5%.

For hindlimb diagnostic analgesia, the highest leave-one-out LDA-classification accuracy (56.1%) was found with the blocked limb on the inside of the circle on soft ground, followed by straight-line trot (51.1%). The lowest leave-one-out classification accuracy was with 34.1%, found with the blocked limb on the outside of the circle when trotting on a soft surface.

### 3.2. Exercise Condition

Figure 2 and Figure 3 illustrate the ability of the LDA-transformed feature space to differentiate between the efficacy categories. Distances between the category centroids (Table 2) provide quantitative insights into the overall distance between the three clusters as well as the distances between pairs of categories.

In agreement with the highest leave-one-out classification accuracy for forelimb diagnostic analgesia, the highest overall distance between efficacy categories (6.13) was achieved for trot on a hard surface with the blocked limb on the inside of the circle. Figure 2F shows generally good separation between the three category clusters, with little overlap. The lowest overall distance (2.23) was found for straight-line trot, almost a factor of three less than for the condition with the highest inter-category distance. Further analysis, shown in Table 2, indicates that trotting with the blocked limb on the inside of the circle on a hard surface provides the highest pairwise inter-category distances for differentiating between partially positive and positive blocks as well as between negative and positive blocks. The highest pairwise inter-category distance between negative and partially positive forelimb DA was found with the blocked limb on the outside of the circle on a hard surface.

In agreement with the highest leave-one-out classification accuracy for hindlimb diagnostic analgesia, the highest overall distance between hindlimb block efficacy categories (5.80) was achieved for trot on a soft surface with the blocked limb on the inside of the circle. Figure 3C shows good separation between the three category clusters. In analogy to the forelimb data, the lowest overall distance (3.41) was found for the straight-line trot. Further analysis, see Table 2, indicates that trotting with the blocked limb on the inside of the circle on a soft surface provides the highest pairwise inter-category distance for differentiating between partially positive and positive blocks. The highest pairwise inter-category distance between negative and partially positive forelimb blocks was found for the average lunge assessment on the soft surface and for the average lunge assessment on the hard surface between negative and positive forelimb blocks.

### 3.3. Movement Asymmetry Parameters

#### 3.3.1. Forelimb Diagnostic Analgesia

**Figure 4:**
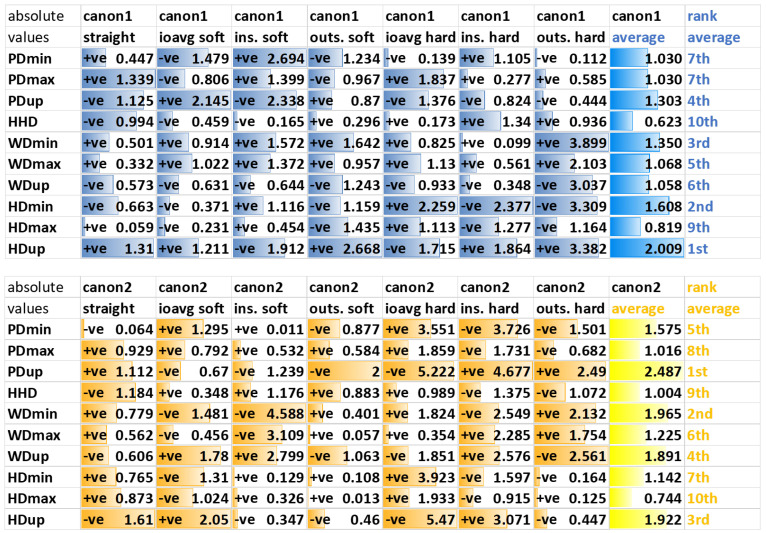
Absolute values of standardized canonical discriminant function coefficients for LDA applied to differences in ten upper body movement asymmetry parameters before/after forelimb diagnostic analgesia. Absolute values are used to create bar graphs illustrating the weighting of all features within the first (canon1) and within the second (canon2) canonical discriminate function. Labels “+ve” and “−ve” indicate the sign of the original values before taking absolute values for illustrative purposes. Ranking of movement symmetry features within the average absolute values (column average) from highest (1st) to lowest (10th) is given in the last column (ranking). The highest total value (one fore forelimb blocks, one for hindlimb blocks), the best separation between subjectively judged diagnostic analgesia efficacy categories is indicated in red, and the lowest values, worst separation, is indicated in green.

Generally, there is a high level of variation in the distribution of the absolute values of the first canonical discriminant function across the ten features across the seven exercise conditions calculated for forelimb diagnostic analgesia (Figure 4).

Along the first canonical function, two head asymmetry parameters, HDmin and HDup, overall show the highest weighting, followed by a withers (WDmin) and then a pelvic (PDup) feature. For straight-line assessment, the first, third and forth highest weightings are attributed to pelvic movement asymmetry parameters (PDmax, PDup, HHD), with the second highest weighting attributed to a head movement asymmetry parameter (HDup). For lunge assessments, head movement asymmetry parameters appear to receive higher weightings on the hard surface, and pelvic movement asymmetry parameters on the soft surface.

Along the second canonical function, HDup is the only head movement feature in the top 6 in the average weighting across exercises. HDup also gets the highest weighting on the straight, where again three of the four pelvic parameters are in the top four (HHD, PDup, PDmax).

#### 3.3.2. Hindlimb Diagnostic Analgesia

**Figure 5:**
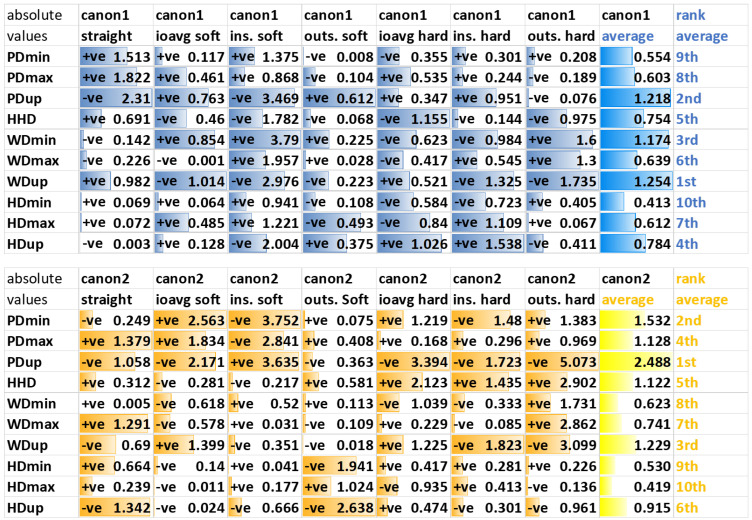
Absolute values of standardized canonical discriminant function coefficients for linear discriminant analysis (LDA) applied to differences in ten upper body movement asymmetry parameters before/after hindlimb diagnostic analgesia. These values are used to create bar graphs (presented in the figure above) illustrating the weighting of asymmetry values within the first (canon1) and the second (canon2) canonical discriminant function. Labels “+ve” and “−ve” are indicating the sign of the values. Ranking of movement symmetry features within the average absolute values (column average) from highest (1st) to lowest (10th) is given in the last column (ranking).

Generally, there is a high level of variation in the distribution of the absolute values of the first canonical discriminant function across the ten movement asymmetry parameters across the seven exercise conditions calculated for hindlimb diagnostic analgesia (Figure 5).

Along the first canonical function, averaged across all exercises, two withers (WDmin and WDup) and one pelvic movement asymmetry parameter (PDup) are assigned the highest weighting. For differentiation between block efficacy categories based on straight-line data, three pelvic movement asymmetry parameters (PDup, PDmax, PDmin) followed by a withers movement asymmetry parameter (WDup) are contributing, with the highest weighting. Except for the hard surface circle assessments, PDup is consistently within the top three weightings.

Along the second canonical function, averaged across all exercises, the four pelvic movement asymmetry parameters and one withers movement asymmetry parameter (WDup) are assigned the highest weightings. With the exception of straight-line trot and lunge exercise on the soft surface with the blocked limb on the outside (where HDmin and HDup are in the top five weightings), head movement asymmetry parameters are assigned low weightings.

## 4. Discussion

In this study, we have investigated the association between the subjectively judged efficacy of diagnostic analgesia and quantitative changes in movement asymmetry.

Linear discriminant analysis (LDA) provides the ability to reduce the dimensionality of a data-driven classification by transforming the original feature space into a set of transformed features that maximize the distance between a set of defined classes. It also provides a framework for expressing the classification performance, for calculating distances between classes in a comparable manner between datasets and is a general method used in pattern recognition for differentiating between multiple categories in the presence of sources of variation [34,35]. It has also been applied previously to scoring hindlimb lameness from IMU data [26]. This has allowed us to compare different exercise conditions and investigate the importance of the original features—derived from head, withers and pelvic movement—in the transformation and classification process. In this first study, we were not interested in further analyzing the detailed influence of potential sources of variation (individual clinician, horse, source of lameness or specific diagnostic block) but rather in investigating the discriminative power of LDA for common exercises and how the exercises highlight which movement asymmetry features are essential in this data-driven process. With increasing amounts of quantitative data collected from many more clinical cases, additional sources of variation will become more important for decision-making about individual patients, and will then need detailed analysis.

### 4.1. Classification Accuracy

For forelimb diagnostic analgesia, both the highest and lowest accuracies for the leave-one-out classification tasks were found for lunge assessment on the hard surface, with 57.4% on the inside rein and 38.3% on the outside rein. In analogy, for hindlimb diagnostic analgesia, both the highest and lowest accuracies for the leave-one-out classification tasks were found for lunge assessment, however, on the soft surface, with 56.1% on the inside rein and 34.1% on the outside rein. The low accuracy, in particular when exercising with the blocked limb on the outside of the circle, may be related to the effect that circle-induced and lameness-induced movement asymmetries cancel each other out [24,37] and that, as such, asymmetries may be small and likely more difficult to perceive [18]. However, this aspect needs to be investigated further because, in the present study, diagnostic analgesia efficacy was not assigned individually to each exercise condition, but rather an overall efficacy value was assigned to all exercises assessed for the evaluated block and subjective ‘labels’ used to reflect the expert opinion of the veterinarians working up the lameness case. Additionally, the variations in accuracy might be related to specific lameness types appearing to be exacerbated depending on the surface or the positioning of the affected limb on the inside or the outside of the circle. For example, lameness in horses with suspensory desmitis is exacerbated on deep, soft surfaces and is often worse with the affected limb on the outside of the circle, and this injury is seen much more commonly in hindlimbs compared to forelimbs [3].

Generally, the present study provided no supporting evidence for the hypothesis that LDA-based classification accuracy for differentiating between different subjective efficacy categories would be higher for forelimb than for hindlimb diagnostic analgesia. The ranges of reclassification and leave-one-out classification accuracies across exercises given in Table 1 are similar between forelimb and hindlimb data. This is surprising in as much as that higher levels of observer agreement have been reported for forelimb lameness scoring compared to the scoring of hindlimb lameness [28]. In the context of a data-driven classification, the high variability in asymmetry measures found for head movement among horses and between reins [19,38] contributes to higher levels of ‘noise’ in the data likely impeding automated classification. It should also be noted that the comparatively high differences between reclassification and leave-one-out classification accuracy reported in the present study (Table 1) indicate that some of the LDA transformations may not generalize well across the data subsets. In particular, for the individual-rein data with smaller sample sizes (N = 41 to N = 58), differences of up to 34% are found between reclassification and leave-one-out classification. In contrast, for straight-line and combined-surface data, smaller differences of 9–17% are reported with larger sample sizes between N = 86 and N = 99.

### 4.2. Exercise Condition

Scatter plots and distances between block efficacy category centroids confirmed that lunge data are contributing in a useful manner to the data-driven differentiation between subjective block efficacies (Figure 2 and Figure 3).

#### 4.2.1. Forelimb Diagnostic Analgesia

Exercising with the blocked limb on the inside of the circle on a hard surface appears particularly advantageous and provides the highest inter-centroid difference (Figure 2, Table 2). This exercise condition also shows the highest values for two of the distances between pairs of centroids (between partially positive and positive as well as between negative and positive forelimb blocks; Table 2). That circling on a hard surface is beneficial should not come as a surprise, based on clinical experience [3] but also because head movement asymmetry increases on a hard surface compared to straight-line exercise [21]. However, there is a considerable amount of variation between horses and reins in relation to changes in head movement on the lunge [23,24]. Investigating potential confounding variables such as the specific block administered within a limb may aid in better understanding the source of these variations. Specific LDA transformations for specific types of diagnostic analgesia may improve classification performance in the future.

#### 4.2.2. Hindlimb Diagnostic Analgesia

The highest inter-centroid difference was reported for lunge exercise with the blocked limb on the inside of the circle (Table 2, Figure 3). However, in contrast to forelimb diagnostic analgesia, distances between subjective efficacy centroids were highest for soft surface exercise with the blocked limb on the inside of the circle. This exercise condition also provides the highest distance between partially positive and positive hindlimb blocks (Table 2).

#### 4.2.3. Average across Reins

For both forelimb and hindlimb data, inter-centroid distances were smaller for straight-line exercise than for any of the lunge exercises. In agreement with clinical practice [3], this highlights the usefulness of exercising horses in circles from a data-driven decision-making perspective.

For hindlimb data, the highest inter-centroid distances between negative and partially positive as well as between negative and positive blocks were found for average-rein movement asymmetry data. This is particularly interesting in light of a previous study (without diagnostic analgesia) which reported smaller average-rein asymmetry values across reins compared to straight-line asymmetry values [36]. Despite that, the higher inter-centroid differences between efficacy categories for average-rein data indicate that differences in movement asymmetry before/after hindlimb diagnostic analgesia may increase data consistency and remove ‘noise’ from LDA-based decision making. It should also be noted that the circle radius was not tightly controlled in this multi-center study and that differences in circle radius and speed have been shown to influence movement symmetry [20,39]. This should be kept in mind when combining data from left and right reins.

For forelimb data, on the other hand, average-rein data appeared less beneficial, with the highest inter-centroid distances all reported for individual-rein data. This may be related to the higher variation between horses and reins for head movement [23,24] and indicates, in contrast to hindlimb data, that average-rein data cannot effectively remove this ‘noise’.

### 4.3. Asymmetry Parameters

When studying the ranking of the weightings attributed to each of the ten asymmetry parameters across exercise conditions and across differentiating between efficacy categories for both forelimb and hindlimb diagnostic analgesia (Figure 4 and Figure 5), the top three parameters are HDup (1st and 4th), PDup (4th and 2nd) and WDmin (3rd and 3rd). This highlights the fact that movements across the whole body from the head, via the withers, to the sacrum need to be considered when judging the effect of diagnostic analgesia and reinforces recent studies reporting that withers movement is important for quantifying lameness [40,41,42].

In agreement with our third hypothesis, two (of three) head movement parameters receive the first and second highest weighting across forelimb LDAs, emphasizing the importance of the head nod in the context of forelimb lameness [43]. One pelvic movement parameter (PDup) is among the top three highest weightings for hindlimb LDAs, providing some supporting evidence for our third hypothesis in relation to hindlimb diagnostic analgesia. However, withers movement asymmetry (WDup and WDmin) appears at least equally important for data-driven categorization of the success of hindlimb DA, emphasizing the cross-body complexity of movement patterns [40,41] beyond the documented hip or pelvic hike [44].

Movement asymmetry changes after diagnostic analgesia confirm the veterinary “law-of-sides”, indicating that horses with a true hindlimb lameness show head movement mimicking an ipsilateral forelimb lameness and that horses with a true forelimb lameness show pelvic movement mimicking a contralateral hindlimb lameness [45,46]. Some supporting evidence for the “law-of-sides” can be found in the present study: for example, the weightings for PDup and HDup for the first canonical function of all seven hindlimb LDAs (Figure 5) agree, indicating ipsilateral changes in these asymmetry parameters, as expected for changes after hindlimb DA [45]. However, in contrast to this finding, movement asymmetry changes can be more complex on the circle than what the “law-of-sides” suggests [37].

The compensatory effect is also less clear for forelimb DA (Figure 4): Three exercises (straight line, outside soft, outside hard) show evidence of the expected contralateral effect, i.e., a positive weighting for HDup paired with a negative weighting for PDup in agreement with the “law-of-sides”. The other four exercise conditions are showing evidence of an ipsilateral effect between changes in HDup and PDup. There is previous conflicting evidence that compensatory movements in forelimb lameness are inconsistent between horses [47], that there are both ipsilateral and contralateral compensatory components during circular movement [37] and that specifically the pushoff phase of the contralateral hindlimb is affected [46].

Withers movement has been shown to provide relevant information for differentiating between a true forelimb lameness and a compensatory head nod in reaction to a hindlimb lameness [40,41]. The fact that one withers parameter (WDmin) receives the third highest weighting within the first canonical discriminant function for both forelimb and hindlimb LDAs (averaged across exercises) highlights the general importance of withers movement asymmetry for data-driven categorization of changes of diagnostic analgesia. However, the relationships between the signs of corresponding head and withers parameters, for example between HDmin and WDmin or between HDup and WDup (Figure 4 and Figure 5), do not provide evidence for consistent differentiation between forelimb and hindlimb diagnostic analgesia.

Similar to studies conducted for straight-line movement [15,16,48,49], it appears beneficial to quantify the contralateral force distribution between pairs of limbs in lame horses on the lunge. This might help uncover fundamental mechanisms governing compensatory movement patterns across exercises.

### 4.4. Limitations

In addition to the documented expectation bias [17] when subjectively judging the effect of diagnostic analgesia, the present study was conducted at two veterinary hospitals routinely employing quantitative gait analysis during lameness examinations. As a consequence, the clinicians judging the efficacy of diagnostic analgesia have had access to quantitative information in the form of asymmetry parameters during the workup. This will likely have influenced their subjective judgement; a subjective assessment blinded to the outcomes of gait analysis would have provided two truly independent sources of information. There are some considerations that appear particularly relevant in this context.

First, the gait analysis system used in this study provides data with a delay of 30 s or more after the horse has finished a straight-line exercise and of around 60 s for longer exercises such as lunging. Additional time is then needed to create left-right rein average data or comparison plots. This general delay until the gait analysis results are available provides the clinicians time to consider their subjective opinion about the efficacy of the block. This may reduce, but does not completely eliminate, the influence of gait analysis results in the decision-making process about block efficacy.

Second, the current guideline values of 6 mm for head movement and 3 mm for pelvic movement asymmetry [14] (adjusted to 8 mm and 5 mm, respectively, for the system used in this study [50]), are only directly applicable to straight-line data because inter-measurement variability has been documented to be higher on the lunge [19]. The lack of relevant guideline values for anything but straight-line data limits (but again does not eliminate) the influence of quantitative gait data for the subjective judgement.

Third, contrary to clinical practice whereby individual exercise conditions are assigned different lameness grades [3], our approach here was to assign one ‘global’ efficacy category across all the exercises based on retrospective consultation of the clinical case records where terms such as ‘negative response’, ‘partially positive response’ or ‘positive response’ were mentioned for specific blocks or subjective impressions about the amount of improvement after a block were given in percentage numbers which were mapped to the categories as described. The process of using a ‘global’ efficacy score was implemented to limit the ‘noise’ (uncertainty) in the subjective data, i.e., the documented variabilities in inter-observer agreement for lameness grading [28,51,52], and specific difficulties when visually assessing lameness on the lunge [37]. There are currently no quantitative studies on the effect of inter-observer variation in evaluating the efficacy of diagnostic analgesia for lunge exercise.

It appears crucial that future studies with larger datasets investigate inter-observer variation as an important source of variation and consider the subjective judgement of each exercise condition in relation to, for example, specific origins of lameness, the number and order of blocks applied and the number of limbs involved in a lameness. This appears crucial in the context of documented challenges encountered in human decision making based on multiple parameters [53]. Put simply, the aim of the current study was to provide some limited, first insights into the fundamental ‘mechanisms’ by employing a robust, basic data-driven method to ‘real-life’ data including a number of sources of variation. The reader is firmly encouraged to take into account the limitations of the chosen approach when drawing conclusions about the individual exercise conditions.

## 5. Conclusions

Accuracy of data-driven classification of changes in gait asymmetry after diagnostic analgesia varied considerably for both forelimb and hindlimb data, likely reflecting the variable responses of lame horses across exercise conditions.

The best separation between categories was measured with the blocked limb on the inside of the circle, highlighting the usefulness of lunge exercise and in particular consulting quantitative gait data of this exercise condition before/after diagnostic analgesia, on hard ground for horses with forelimb lameness and on soft ground for horses with hindlimb lameness.

Movement asymmetry parameters from across the body—derived from vertical movement of head, withers and pelvis—received high weightings within the first canonical LDA functions across exercises. This highlights that movement changes after diagnostic analgesia affect the whole upper body and that compensatory movement changes should be considered when quantitative gait data for changes before/after diagnostic analgesia.

## Figures and Tables

**Table 1:** Reclassification and leave-one-out classification accuracy after linear discriminant analysis (LDA) transformation. Presented are results for two data subsets (forelimb diagnostic analgesia DA: F; hindlimb DA: H) of movement asymmetry differences before/after administration of diagnostic analgesia in N = 53 horses. Classification results are given for straight-line, inside rein, outside rein and the average rein conditions (avg) as well as a further subdivision by surface (soft or hard), where applicable. Weighted averages are calculated taking into account the sample sizes for each condition. Highest values, the best separation between subjectively judged diagnostic analgesia efficacy categories (from retrospective consultation of case records), are indicated in red, and the lowest values, worst separation, are indicated in green.

Fore/ Hind	Direction	Surface	Reclass. (%)	Leave-1-Out (%)	Abs Diff. (%)	N
**F**	**str**	**Hard**	**53.5**	**39.5**	**14**	**86**
**F**	**Avg**	**All**	**63.6**	**52.5**	**11.1**	**99**
		Soft	63.5	46.2	17.3	52
		Hard	80.9	51.1	29.8	47
**F**	**Inside**	**All**	**63.6**	**50.5**	**13.1**	**99**
		Soft	57.7	40.4	17.3	52
		Hard	68.1	57.4	10.7	47
**F**	**Outside**	**All**	**61.6**	**52.5**	**9.1**	**99**
		Soft	63.5	55.8	7.7	52
		Hard	72.3	38.3	34	47
***F***	***weighted total***		***60.82***	***49.06***		***383***
**H**	**Str**	**Hard**	**60.2**	**51.1**	**9.1**	**88**
**H**	**Avg**	**All**	**60.6**	**49.5**	**11.1**	**99**
		Soft	70.7	46.3	24.4	41
		hard	60.3	48.3	12	58
**H**	**Inside**	**all**	**61.6**	**44.4**	**17.2**	**99**
		soft	**78**	**56.1**	21.9	**41**
		hard	62.1	44.8	17.3	58
**H**	**Outside**	**all**	**51.5**	**38.4**	**13.1**	**99**
		soft	68.3	34.1	34.2	41
		hard	58.6	39.7	18.9	58
***H***	***weighted total***		***58.43***	***45.70***		***385***

**Table 2:** Distances between category centroids of ‘negative’, ‘partially positive’ and ‘positive’ forelimb diagnostic analgesia (DA) based on canonical discriminate functions calculated through linear discriminant analysis for seven different exercise conditions for features quantifying differences in movement asymmetry before/after forelimb and hindlimb diagnostic analgesia: neg2part, distance between centroids of negative and partially positive blocks; part2pos, distance between centroids of partially positive and positive blocks; neg2pos, distance between centroids of negative and positive blocks; Highest Indiv., highest distance between block categories with respect to which exercise condition it was recorded for. Highest values, the best separation between subjectively judged diagnostic analgesia efficacy categories (from retrospective consultation of case records), are indicated in red, and the lowest values, worst separation, are indicated in green; separately for forelimb and hind limb diagnostic analgesia.

Blocked Limb	Direction	Surface	neg2part	part2pos	neg2pos	Total	Highest Indiv.
**F**	str	hard	0.65	0.62	0.96	**2.23**	
	avg	Soft	1.64	1.14	1.49	**4.27**	
		Hard	1.30	2.34	1.91	**5.56**	
	inside	Soft	1.23	0.67	1.31	3.21	
		Hard	1.36	**2.48**	**2.29**	6.13	part2pos, neg2pos
	outside	Soft	1.39	1.68	1.33	4.39	
		Hard	**1.80**	2.03	1.10	4.93	neg2part
**H**	str	Hard	0.96	1.07	1.38	**3.41**	
	avg	Soft	**2.05**	1.48	1.28	**4.81**	neg2part
		Hard	0.95	1.69	**2.03**	**4.67**	neg2pos
	inside	Soft	2.03	**2.06**	1.71	5.80	part2pos
		Hard	0.88	1.51	1.69	4.08	
	outside	Soft	1.75	1.08	1.53	4.37	
		Hard	1.11	1.30	1.47	3.89	

## Data Availability

Data are available via figshare at https://doi.org/10.6084/m9.figshare.19105016.v2 (accessed on 4 February 2022).

## Supplementary Materials

The following supporting information can be downloaded at: https://www.mdpi.com/article/10.3390/ani12060762/s1, Table S1: Difference in normalized vertical head, withers and pelvic movement symmetry before/after diagnostic analgesia in 53 horses undergoing veterinary lameness examinations during straight-line trot and trot on the lunge on hard and/or soft ground.
